# Linkages between HIV-1 specificity for CCR5 or CXCR4 and *in vitro* usage of alternative coreceptors during progressive HIV-1 subtype C infection

**DOI:** 10.1186/1742-4690-10-98

**Published:** 2013-09-16

**Authors:** Kieran Cashin, Martin R Jakobsen, Jasminka Sterjovski, Michael Roche, Anne Ellett, Jacqueline K Flynn, Katharina Borm, Maelenn Gouillou, Melissa J Churchill, Paul R Gorry

**Affiliations:** 1 Center for Biomedical Research, Burnet Institute, 85 Commercial Rd, Melbourne, Victoria 3004, Australia; 2 Center for Population Health, Burnet Institute, Melbourne, Australia; 3 Department of Microbiology and Immunology, University of Melbourne, Melbourne, Australia; 4 Department of Biomedicine, Aarhus University, Aarhus, Denmark; 5 Department of Infectious Diseases, Monash University, Melbourne, Australia; 6 Department of Microbiology, Monash University, Melbourne, Australia; 7 Department of Medicine, Monash University, Melbourne, Australia; 8 Department of Microbiology, La Trobe University, Melbourne, Victoria, Australia

**Keywords:** HIV-1, Env, Subtype C, CCR5, CXCR4, Alternative coreceptor, Pathogenesis

## Abstract

**Background:**

Human immunodeficiency virus type 1 (HIV-1) subtype C (C-HIV) is spreading rapidly and is now responsible for >50% of HIV-1 infections worldwide, and >95% of infections in southern Africa and central Asia. These regions are burdened with the overwhelming majority of HIV-1 infections, yet we know very little about the pathogenesis of C-HIV. In addition to CCR5 and CXCR4, the HIV-1 envelope glycoproteins (Env) may engage a variety of alternative coreceptors for entry into transfected cells. Whilst alternative coreceptors do not appear to have a broad role in mediating the entry of HIV-1 into primary cells, characterizing patterns of alternative coreceptor usage *in vitro* can provide valuable insights into mechanisms of Env-coreceptor engagement that may be important for HIV-1 pathogenesis.

**Results:**

Here, we characterized the ability of luciferase reporter viruses pseudotyped with HIV-1 Envs (n = 300) cloned sequentially from plasma of 21 antiretroviral therapy (ART)-naïve subjects experiencing progression from chronic to advanced C-HIV infection over an approximately 3-year period, who either exclusively maintained CCR5-using (R5) variants (n = 20 subjects) or who experienced a coreceptor switch to CXCR4-using (X4) variants (n = 1 subject), to utilize alternative coreceptors for entry. At a population level, CCR5 usage by R5 C-HIV Envs was strongly linked to usage of FPRL1, CCR3 and CCR8 as alternative coreceptors, with the linkages to FPRL1 and CCR3 usage becoming statistically more robust as infection progressed from chronic to advanced stages of disease. In contrast, acquisition of an X4 Env phenotype at advanced infection was accompanied by a dramatic loss of FPRL1 usage. Env mutagenesis studies confirmed a direct link between CCR5 and FPRL1 usage, and showed that the V3 loop crown, but not other V3 determinants of CCR5-specificity, was the principal Env determinant governing the ability of R5 C-HIV Envs from one particular subject to engage FPRL1.

**Conclusions:**

Our results suggest that, in the absence of coreceptor switching, the ability of R5 C-HIV viruses to engage certain alternative coreceptors *in vitro*, in particular FPRL1, may reflect an altered use of CCR5 that is selected for during progressive C-HIV infection, and which may contribute to C-HIV pathogenicity.

## Background

Entry of human immunodeficiency virus type 1 (HIV-1) into cells involves the interaction of the viral gp120 envelope glycoproteins (Env) with cellular CD4 and a secondary coreceptor, which is typically one of the chemokine receptors CCR5 or CXCR4 [1]. HIV-1 Envs are phenotypically characterized by their ability to use CCR5 (R5), CXCR4 (X4) or both coreceptors (R5X4) for entry. In addition to CCR5 and CXCR4, alternative coreceptors such as CCR1, CCR2, CCR3, CCR8, CX3CR1, CXCR6, FPRL1, GPR1, GPR15, APJ, STRL33 and D6 can act as lentiviral coreceptors and mediate the entry of certain HIV-1, HIV-2 and simian immunodeficiency virus (SIV) strains into transfected cell lines [2-5].

Whilst alternative coreceptors do not appear to have a broad role in mediating the entry of HIV-1 into primary cells, a recent report described acute HIV-1 infection with a variant that could not use CCR5 or CXCR4, and used only GPR15 efficiently [6], suggesting a larger potential role for alternative coreceptors than currently recognized. SIV strains do not use CXCR4, but exhibit alternative coreceptor usage that is typically broader and more efficient than HIV-1 subtype B (B-HIV) [2,7]. HIV-1 subtype C strains (C-HIV) also rarely use CXCR4, although CXCR4 usage may be more common than previously appreciated (reviewed in [8,9]), and have been reported to have relatively efficient *in vitro* usage of the alternative coreceptors CCR3, CCR8 and FPRL1 [10,11], or GPR15, CXCR6 and APJ [12]. Unlike HIV-1, however, non-pathogenic SIV infection in natural hosts can be mediated by CXCR6, GPR15 and GPR1 *in vivo*[13,14]. Similarly, earlier studies showed that red-capped mangabeys usually lack functional CCR5, and that CCR2 was commonly used by SIVrcm strains [15]. These studies suggest an *in vivo* role for alternative coreceptors in SIV infection of natural hosts.

R5 HIV-1 viruses are typically associated with HIV-1 transmission and establishment of new infections, and are dominant in the chronic phase of infection. However, in up to 40 to 50% of individuals infected with B-HIV, progression to late stages of infection is associated with a switch in coreceptor specificity, with emergence of X4 or R5X4 viral variants [16,17]. The emergence of CXCR4-using HIV-1 viruses is associated with rapid CD4+ T-cell decline and progression from chronic to advanced stages of HIV-1 infection. In contrast, most individuals infected with C-HIV, which is the most prevalent HIV-1 subtype worldwide, progress from chronic to advanced stages of infection whilst exclusively harbouring R5 viruses [8,9,18]. Whether disease progression in the presence of only R5 strains reflects mainly cumulative damage of ongoing replication, or indicates the emergence of variants with unique biological features that may contribute to increased pathogenicity is an important question. In support of the latter possibility, recent studies have shown that compared to transmitted/founder (T/F) viruses, R5 viruses from chronic C-HIV infections exhibit a more flexible recognition of CCR5, as demonstrated by their ability to interact with an altered conformation of CCR5 induced by the CCR5 antagonist maraviroc (MVC) [19,20]. In addition, the ability of C-HIV Envs to interact with CCR5 has been shown to correlate with their ability to use CCR3, CCR8 and FPRL1 as alternative coreceptors *in vitro*[10]. Thus, the ability of R5 Envs to engage certain alternative coreceptors, albeit in cell lines, may also reflect an altered use of CCR5 that is selected for during progressive C-HIV infection and which may contribute to C-HIV pathogenicity.

Relatively little is known about the pathogenesis of C-HIV. This is, in part, because nearly all studies on C-HIV have been cross-sectional studies of chronically-infected subjects, studies of early/acute infected individuals, relatively small studies of late stage C-HIV infection where subjects were antiretroviral therapy (ART)-experienced which likely altered the natural history of the disease, or studies which relied on primary C-HIV isolates where passage in PBMC may have resulted in a selection bias [10,12,21-31]. Detailed, longitudinal studies of C-HIV evolution from chronic to advanced stages of infection in clinically well-characterised ART-naïve patients are essential for understanding the importance of Env alterations in C-HIV pathogenesis. Here, we utilized a large panel of functional HIV-1 Envs (n = 300) cloned directly from longitudinally-collected plasma samples of 21 antiretroviral therapy (ART)-naïve subjects from rural Zimbabwe, who experienced progression from chronic to advanced stages of C-HIV infection over an approximately 3 year period [8]. In this cohort, only one subject experienced a coreceptor switch whereby X4 strains emerged from antecedent R5 viruses at late stage infection, with nearly all subjects (n = 20) harbouring only R5 viruses from chronic to advanced infection [8]. Thus, the use of this longitudinal C-HIV Env panel offered a unique opportunity to elucidate whether Env variants are selected for during progressive C-HIV infection that exhibit altered coreceptor engagement that is reflected in an increased ability to utilise alternative coreceptors *in vitro*, and whether particular patterns of alternative coreceptor engagement are linked to the preference of the virus for CCR5 or CXCR4.

## Results and discussion

### High frequency of alternative coreceptor usage by C-HIV Envs from ART-naïve subjects who progressed from chronic to advanced infection

Using a panel of 300 C-HIV Envs cloned from longitudinal plasma samples of 21 ART-naïve subjects who experienced progression from chronic to advanced stages of C-HIV infection over an approximately 3 year period, we recently showed that the vast majority of subjects (n = 20) progressed whilst harbouring only R5 Env variants (n = 294 Envs), suggesting that R5 C-HIV strains evolve *in vivo* in the absence of coreceptor switching [8]. Moreover, a recent cross sectional study showed a functional linkage between the use of CCR5 and the alternative coreceptor FPRL1 that was unique to C-HIV Envs, as well as less robust linkages between CCR5/CCR3 usage and CCR5/CCR8 usage [10], suggesting an altered use of CCR5 by C-HIV Envs that may manifest as greater promiscuity for ability to use certain alternative coreceptors *in vitro*.

**Figure 1:**
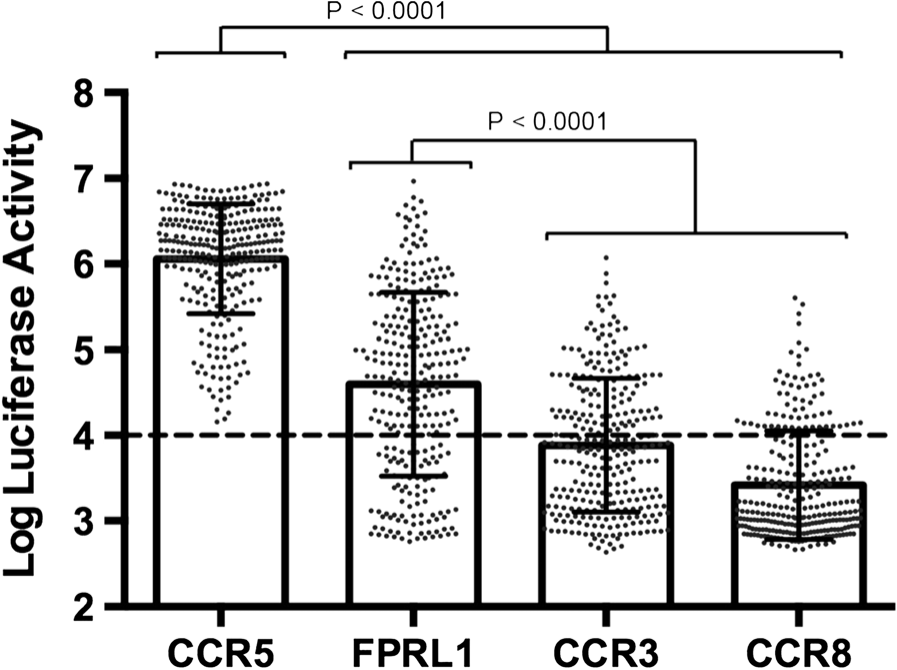
**Alternative coreceptor usage by R5 C-HIV Envs.** Luciferase reporter viruses pseudotyped with each of the C-HIV R5 Envs (n = 294) were used to infect NP2-CD4 cells expressing CCR5, FPRL1, CCR3 or CCR8, and the levels of HIV-1 entry were determined as described in the Methods. The background level of luciferase activity, as determined by infections with luciferase reporter virus pseudotyped with the non-functional ΔKS Env [44], is shown by the horizontal dashed line. The data shown are the means and standard deviations of entry levels of all the R5 C-HIV Envs in the different cell types, and are representative of 3 independent experiments, each performed in triplicate. The individual dots represent mean entry levels of triplicates from a representative experiment. Statistical comparisons were made using a Mann–Whitney *U* test. P values < 0.05 were considered statistically significant.

To determine the frequency of FPRL1-, CCR3- and CCR8-usage by the R5 C-HIV Envs from our cohort, we produced luciferase reporter viruses pseudotyped with each of the 294 R5 Envs, and assessed their ability to enter NP2-CD4 cells expressing either CCR5, FPRL1, CCR3 or CCR8 (Figure 1). Consistent with the results of previous studies [10,11], when analyzed as a group the R5 C-HIV Envs used the alternative coreceptors less frequently than CCR5 (p < 0.0001 by a Mann–Whitney *U*-test), and used CCR3 and CCR8 less frequently than FPRL1 (p < 0.001 by a Mann–Whitney *U*-test). However, 79.6% of the Envs tested (n = 234) were able to use at least one of the alternative coreceptors for entry. Of these, 87.2% (n = 204) used FPRL1, 54.7% (n = 128) used CCR3, 28.2% (n = 66) used CCR8, and 17.1% (n = 40) used all three alternative coreceptors for entry. Notably, 5% of Envs (n = 14) used FPRL1 more efficiently than CCR5. The R5 C-HIV Envs that were negative for alternative coreceptor activity remained negative when higher virus inoculums were used (data not shown). This relatively broad usage of FPRL1, CCR3 and CCR8, whilst concordant with a recent cross sectional study of chronic C-HIV Envs [10], was not observed in another study of C-HIV Envs cloned from recently infected subjects which showed relatively frequent usage of GPR15, CXCR6 and APJ for entry but relatively infrequent usage of FPRL1, CCR3 and CCR8 [12]. Together, these results suggest that usage of FPRL1, CCR3 and CCR8 may be a phenotype of R5 C-HIV Envs that develops at chronic stages of infection, and raises the possibility that the efficiency of *in vitro* FPRL1, CCR3 and CCR8 usage may increase from chronic to late stages of C-HIV infection.

### R5 Envs that persist from chronic to advanced C-HIV infection may have more efficient usage of CCR3 and FPRL1 as alternative HIV-1 coreceptors

To determine whether there are functional linkages between the use of CCR5 and that of FPRL1, CCR3 and/or CCR8 during progressive, untreated C-HIV infection, and whether any such linkages are altered from chronic to advanced stages of C-HIV infection, we next directly compared the efficiency of all the R5 C-HIV Envs to enter NP2-CD4 cells expressing CCR5, FPRL1, CCR3 or CCR8 by correlative analysis. In order to produce robust comparisons, we analyzed the entry levels from a common dilution of virus that resulted in luciferase signals that were within the linear range of activity for each of the cell types that were permissive for virus entry, as described in the Methods.

**Figure 2:**
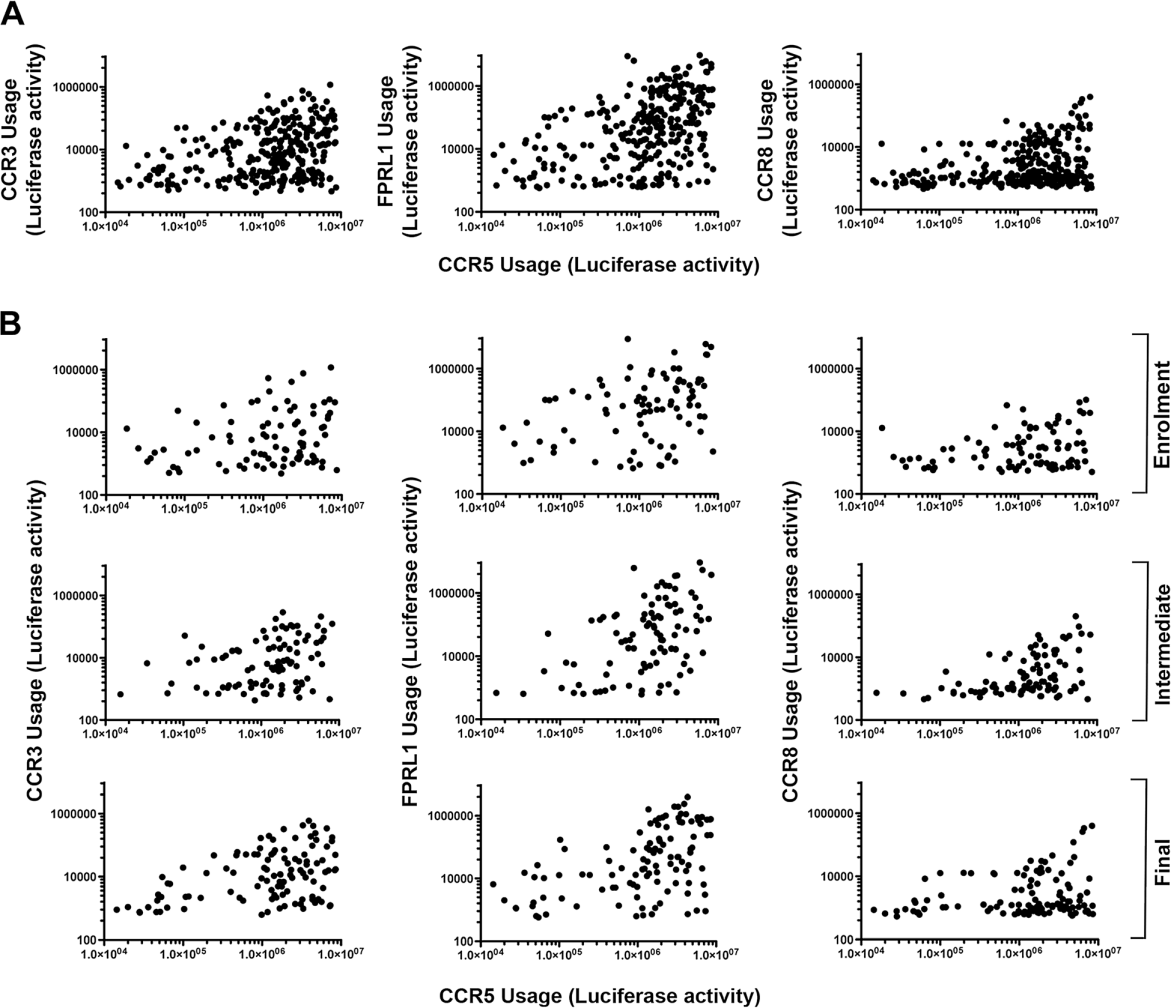
**Associations between CCR5 usage and usage of CCR3, FPRL1 and CCR8 by R5 C-HIV Envs.** Luciferase reporter viruses pseudotyped with each of the C-HIV R5 Envs (n = 294) were used to infect NP2-CD4 cells expressing CCR5, FPRL1, CCR3 or CCR8, and the levels of HIV-1 entry were determined as described in the Methods. Comparative entry levels for each of the individual Envs are shown in Additional file 1: Table S1. **(A)**, scatterplots of all 294 R5 Envs comparing the levels of HIV-1 entry via CCR5 to that of CCR3 (left), FPRL1 (middle), and CCR8 (right). **(B)**, these data were then stratified according to whether the Envs were cloned from the “enrolment” (top row), “intermediate” (middle row) of “final” (bottom row) plasma samples from the longitudinal cohort [8]. Each dot represents the mean value of triplicate experiments. Statistical analysis of these data is shown in Table 1.

The efficiency of the individual C-HIV Envs to use FPRL1, CCR3 and CCR8 as alternative coreceptors for HIV-1 entry, in comparison to their ability to use CCR5 and/or CXCR4, is shown in Additional file 1: Table S1. To determine whether linkages existed between the ability of the R5 Envs to use CCR5 and alternative coreceptors, we first performed univariable clustered linear regression with robust variance for all the Envs across the cohort, regardless of the disease stage. In these analyses, clustered regression was used to account for the possible influence of intra-subject Env similarities. Our results show statistically significant positive correlations between the ability of the R5 C-HIV Envs to use CCR5 and CCR3, FPRL1 and CCR8 (Figure 2A and Table 1). To determine whether these correlations altered during progression from chronic to advanced infection, we next stratified the comparisons according to whether the Envs were cloned from “enrolment”, “intermediate” or “final” plasma samples [8], and re-analyzed the data by univariable clustered linear regression with robust variance (Figure 2B and Table 1). These results show statistically significant correlations between the ability of the R5 C-HIV Envs to use CCR5 and CCR3, FPRL1 and CCR8 at all 3 timepoints, with the exception of the CCR5/CCR3 correlation at the “intermediate” timepoint which neared statistical significance (p = 0.055). Of note, these longitudinal analyses showed near-significant increases over time in the regression coefficients (ie., the slopes of the best fit lines from the scatter plots) for the correlations between CCR5/FPRL1 usage (β increases from 0.232 to 0.336; p = 0.083) and CCR5/CCR3 usage (β increases from 0.222 to 0.417; p = 0.091), but not CCR5/CCR8 usage (β remains unchanged; p = 0.993) (Table 1). Although not quite reaching statistical significance, these results suggest that R5 C-HIV Envs may evolve from chronic to advanced infection to become better able to utilize the alternative coreceptors FPRL1 and CCR3 for HIV-1 entry. It is also possible that CCR5 usage by C-HIV Envs becomes more efficient at late stage infection, which may contribute also to increased FPRL1 and CCR3 usage. Quantitative studies using the 293-Affinofile affinity profiling system and mathematical modeling using VERSA (viral entry receptor sensitivity analysis) metrics [32,33] are in progress to determine if this is the case.

**Table 1:** Statistical analysis of correlations between CCR5 and alternative coreceptor usage

**Correlation**	**All Envs**			**Enrolment Envs**			**Intermediate Envs**			**Final Envs**		
	**β**	**95% CI**	**p-value**	**β**	**95% CI**	**p-value**	**β**	**95% CI**	**p-value**	**β**	**95% CI**	**p-value**
CCR5/FPRL1	0.268	0.178, 0.359	**<0.001**	0.232	0.116, 0.347	**<0.001**	0.228	0.113, 0.343	**0.001**	0.336	0.196, 0.476	**<0.001**
CCR5/CCR3	0.284	0.148, 0.420	**<0.001**	0.222	0.029, 0.384	**0.010**	0.204	-0.005, 0.413	0.055	0.417	0.231, 0.604	**<0.001**
CCR5/CCR8	0.314	0.160, 0.467	**<0.001**	0.304	0.048, 0.561	**0.023**	0.366	0.185, 0.547	**0.001**	0.282	0.076, 0.488	**0.010**

Statistical analysis was conducted by univariable clustered linear regression with robust variance. P values < 0.05 were considered statistically significant.

### The pattern of alternative coreceptor usage by C-HIV Envs appears to be constrained by the primary coreceptor specificity

Although there is accumulating data on the patterns of alternative coreceptor usage by R5 C-HIV Envs [10-12], including our own data from the preceding studies, the repertoire of alternative coreceptor usage by primary X4 C-HIV Envs is unknown. One of the 21 subjects from our cohort (subject 1109) experienced a coreceptor switch to X4 variants that were dominant in the “final” plasma sample, which evolved over a ~3 year interval from antecedent R5 viruses that were dominant in the “enrolment” and “intermediate” plasma samples [8]. Subject 1109 affords an opportunity to examine the evolution of alternative coreceptor usage by C-HIV in the context of primary coreceptor switching.

**Figure 3:**
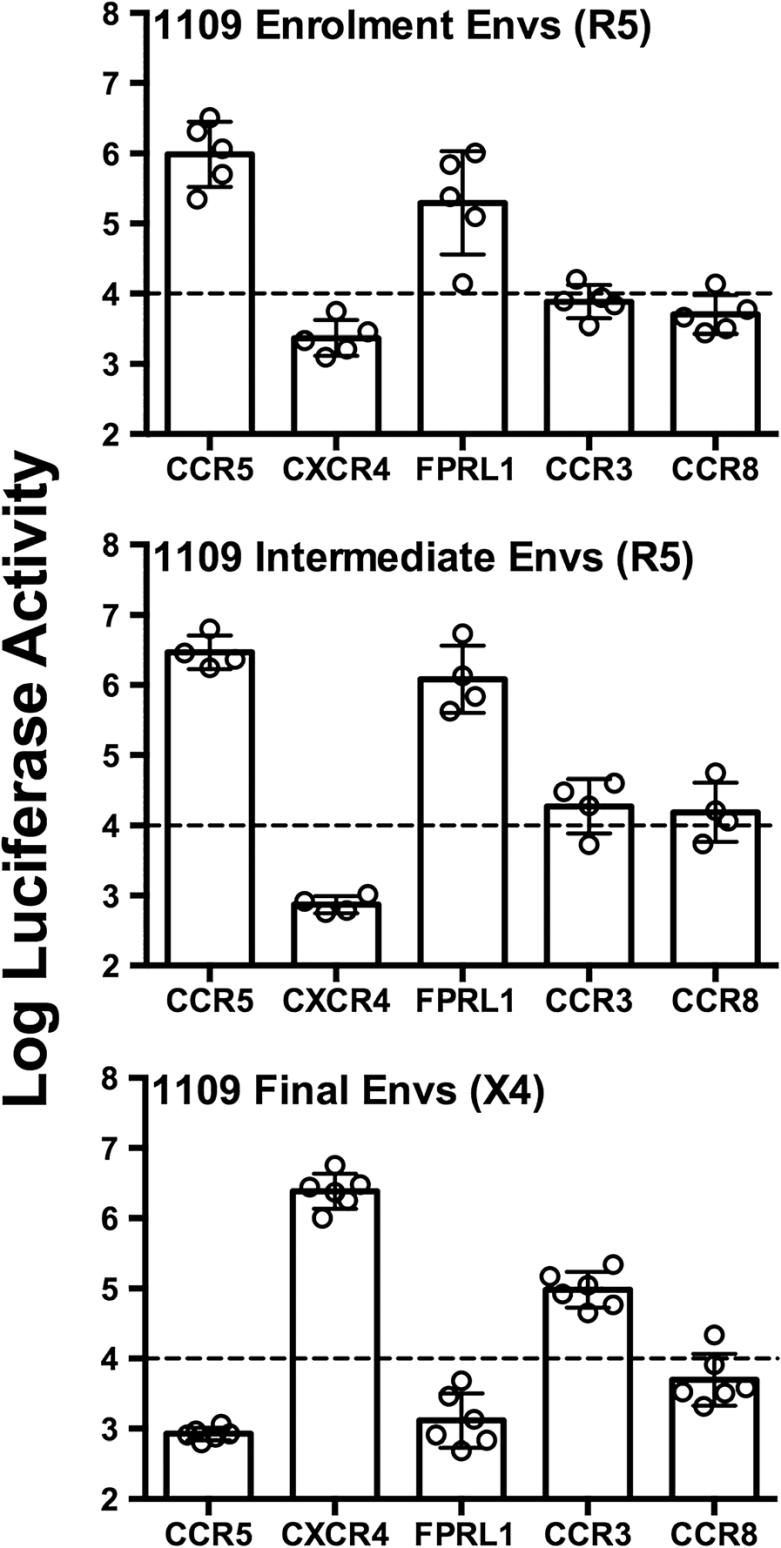
**Changes in alternative coreceptor usage during the transition from R5 to X4 phenotype in subject 1109.** Luciferase reporter viruses pseudotyped with “enrolment” (R5, n = 5), “intermediate” (R5, n = 4) or “final” (X4, n = 6) Envs from subject 1109 [8] were used to infect NP2-CD4 cells expressing CXCR4, CCR5, FPRL1, CCR3 or CCR8, and the levels of HIV-1 entry were determined as described in the Methods. The dotted lines indicate the limit of detection of coreceptor activity, as determined by infections with luciferase reporter virus pseudotyped with the non-functional ΔKS Env [44]. Comparative entry levels for each of the individual Envs are shown in Additional file 1: Table S1. The data shown are means and standard deviations of entry levels in the different cell types, and are representative of 3 independent experiments, each performed in triplicate. The individual circles represent mean entry levels of each of the individual Env clones that were performed in triplicate, from a representative experiment.

We therefore next compared the ability of the “enrolment” R5 (n = 5), “intermediate” R5 (n = 4), and “final” X4 (n = 6) Envs from subject 1109 to utilize CCR5, CXCR4, FPRL1, CCR3 and CCR8 for HIV-1 entry. The efficiency of these individual Envs to utilize the alternative coreceptors in comparison to CCR5 and CXCR4 is shown in Additional file 1: Table S1, and stratified according to disease stage in Figure 3. Our results show that the R5 “enrolment” and “intermediate” Envs have very similar patterns of alternative coreceptor usage, characterized by highly efficient usage of FPRL1 and relatively inefficient usage of CCR3 and CCR8. In contrast, the acquisition of CXCR4 usage by the “final” Envs in subject 1109 was accompanied by a dramatic loss of ability to use FPRL1, a substantial increase in ability to use CCR3, and no change in ability to use CCR8. These studies demonstrate that in the C-HIV variants from subject 1109, a striking inversion of specificity for alternative coreceptors accompanied the switch in primary coreceptor specificity from R5 to X4 phenotype. Interestingly, in a longitudinal analysis of another subject with C-HIV infection, transition from R5 to R5X4 phenotype did not result in loss of FPRL1 usage [11]. Together, these studies further illustrate the functional link between CCR5/FPRL1 usage.

### Mechanistic insights into the linkage between CCR5 and FPRL1 usage by C-HIV Envs

**Figure 4:**
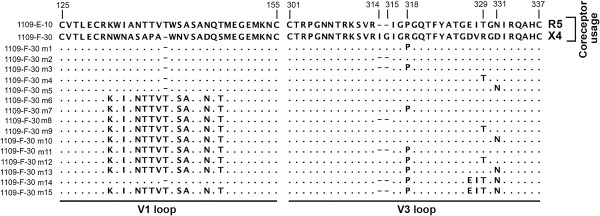
**C-HIV Env mutants.** Amino acid sequences of the Env mutants, aligned against the gp120 sequence of the X4 1109-F-30 sequence. The sequences and descriptions of these Env mutants have been reported previously [8], and are included again here to assist in the interpretation of the functional data. Dots indicate residues identical to 1109-F-30, dashes indicate gaps. Numbers refer to amino acid positions in the V1 and V3 loop regions. Descriptions of the Env mutants are provided in greater detail in the Methods.

**Figure 5:**
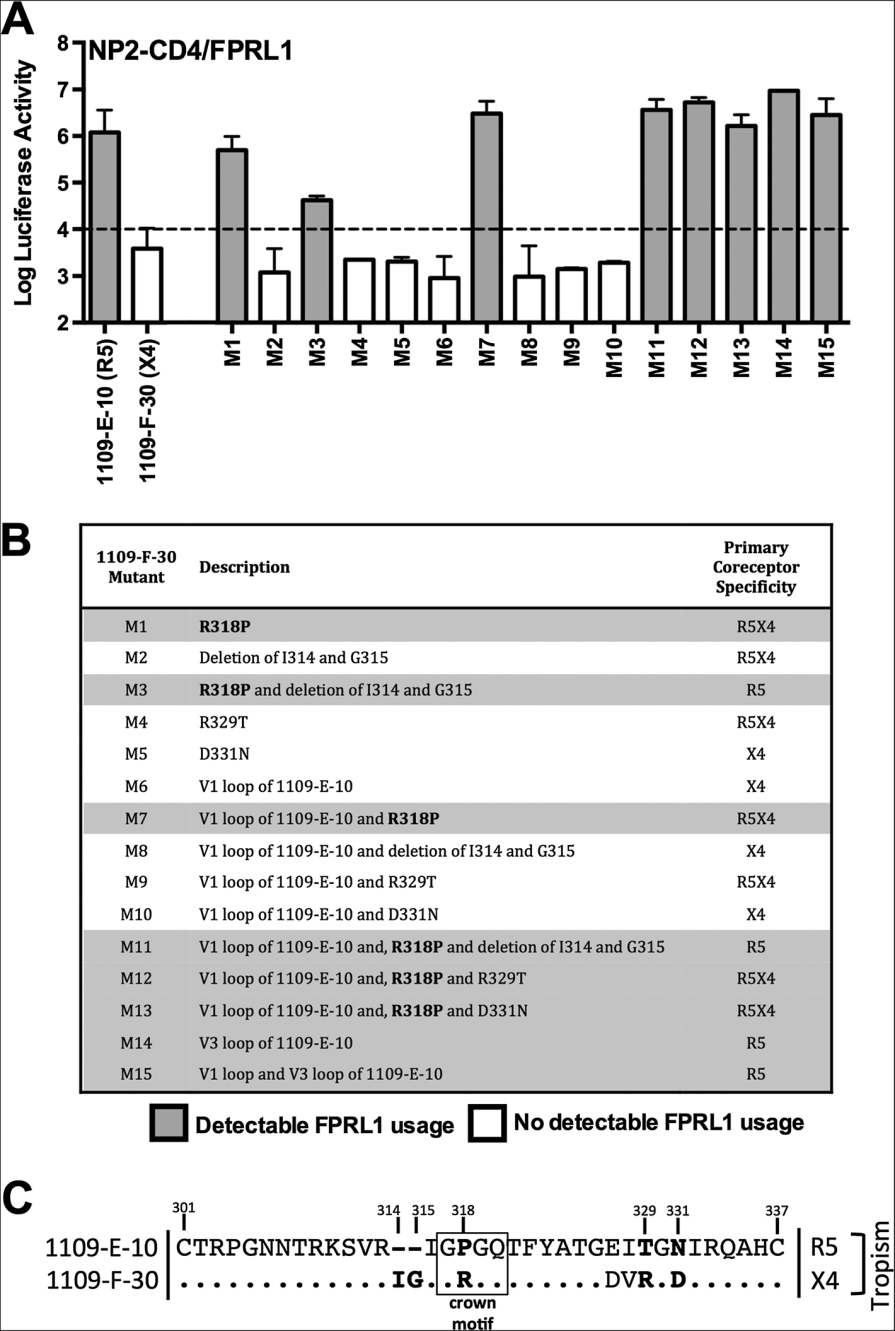
**V3 loop alterations important for the association between CCR5 and FPRL usage. (A)**, luciferase reporter viruses pseudotyped with Env mutants (M1 through M15), or unmodified 1109-E-10 and 1109-F-30 Envs were used to infect NP2-CD4/FPRL1 cells, and the levels of HIV-1 entry were determined as described in the Methods. Open bars indicate Envs where no detectable FPRL1 usage was observed, and shaded bars indicate Envs with detectable FPRL1 usage. The dotted line indicates the limit of detection of coreceptor activity, as determined by infections with luciferase reporter virus pseudotyped with the non-functional ∆KS Env [44]. The results shown are a compilation of 3 independent experiments, each performed in triplicate. The data shown are means, and the error bars represent standard errors of the means. **(B)**, summary of descriptions of the Env mutants. Shaded rows illustrate the Env mutants which had detectable FPRL1 usage. The R318P mutation, which was strongly associated with both CCR5 and FPRL1 usage, is highlighted in bold. The primary coreceptor specificity of the Env mutants (ie., whether they exhibit an R5, R5X4 or X4 phenotype) was determined previously [8]. **(C)**, comparisons of the V3 loop region between the R5 1109-E-10 and X4 1109-F-30 Envs. Shown in bold are the amino acids tested by mutagenesis for modulation of FPRL1 usage, and boxed is the V3 crown motif shown to principally modulate FPRL1 usage.

Whilst at a population level the preceding studies demonstrate significant linkages between the usage of CCR5 and alternative coreceptors by C-HIV Envs, the mechanistic basis for these linkages is unknown. To better understand the underlying Env determinants for these linkages, we used a panel of Env mutants, which we used in our previous studies [8] to define the critical V3 loop changes involved in reverting the X4 phenotype of 1109-F-30 Env to R5X4 intermediates, and to a completely CCR5-restricted phenotype. The amino acid sequences of the Env mutants are shown in Figure 4, and their descriptions and primary coreceptor specificities, which were determined in our recent studies [8], are summarized in Figure 5B.

Luciferase reporter viruses pseudotyped with the Env mutants (M1 through M15) or the unmodified X4 1109-F-30 Env and R5 1109-E-10 Envs were characterized for their ability to enter NP2-CD4/FPRL1 cells (Figure 5A); we chose to conduct these studies in FPRL1-expressing cells but not in CCR3- or CCR8-expressing cells, because only FPRL1 was used by the R5 1109-E-10 Env and not by the X4 1109-F-30 Env. Our results show that efficient use of FPRL1 was restored to the 1109-F-30 Env by the M1, M3, M7, M11, M12, M13, M14 and M15 mutations, but was not restored by the M2, M4, M5, M6, M8, M9 or M10 mutations (Figure 5A,B). These results indicate that changing Arg318 in 1109-F-30 to Pro318, thus restoring the “Gly317-Pro318-Gly319-Gln320” crown to the V3 loop (boxed in Figure 5C), either by itself or in combination with other mutations resulted in efficient FPRL1 usage. In contrast, all of the 1109-F-30 Env mutants which did not use FPRL1 maintained the original Arg318 residue. Together, these results suggest that the gp120 V3 crown is an important determinant for the maintenance of efficient FPRL1 usage by C-HIV strains harbored by subject 1109, and that alteration of the “Gly317-Pro318-Gly319” motif during the transition from R5 to X4 C-HIV variants in this subject abruptly abolishes FPRL1 usage.

Using this panel of mutants we recently showed that abolishing the Ile314-Gly315 insertion present in the X4 1109-F-30 Env (illustrated in Figure 5C), or changing Arg318 to Pro318, resulted in acquisition of CCR5 usage conferring an R5X4 phenotype, and that the presence of both of these changes together abolished CXCR4 usage altogether and conferred an R5 phenotype [8]. Here, we show that just the Arg318 to Pro318 change that is linked to CCR5 usage in this subject is critical for FPRL1 usage. Although limited to a single subject, these results provide a potential mechanistic basis for the linkage between CCR5 usage and FPRL1 usage by C-HIV Envs, which may principally involve the V3 loop crown but not other V3 loop changes associated with specificity for CCR5. Further studies of C-HIV strains isolated from additional subjects are required to more precisely determine the significance of the V3 loop crown in mediating FPRL1 usage.

## Conclusions

The mechanisms underlying the pathogenicity of C-HIV strains remain to be determined by future studies, and indeed, with the establishment of our longitudinal C-HIV Env panel we are now well positioned to learn more about the Env determinants of C-HIV pathogenesis. The observation of unique functional linkages between the ability of R5 C-HIV Envs to use CCR5 and either CCR3, FPRL1 or CCR8 as alternative coreceptors for HIV-1 entry shown here and in previous studies [10] suggests unique selection pressures imposed on C-HIV strains. Our longitudinal analysis of alternative coreceptor usage which suggests an improvement in the ability of R5 Envs to use CCR3 and FPRL1 as alternative coreceptors at late stage infection, and our mutagenesis studies which suggest a critical role for the V3 loop crown in the functional linkage between CCR5/FPRL1 usage, provide additional insights into how R5 C-HIV Envs may evolve *in vivo* and contribute to C-HIV pathogenesis. Since the V3 loop crown interacts with the coreceptor ECLs, these findings suggest that R5 C-HIV Envs may undergo adaptive alterations to acquire an altered and/or more efficient interaction with the CCR5 ECL2 region (that is reflected also in an increased ability to engage the alternative coreceptors), as has been shown recently to occur with certain late-stage, macrophage tropic B-HIV Envs [34]. This could serve to promote increased promiscuity of the Env for the utilization of alternative CCR5 conformations, as has been shown recently for certain chronic C-HIV Envs in comparison to transmission/founder C-HIV Envs [19,20]. Together, these observations support the hypothesis that R5 C-HIV strains may evolve *in vivo* via altered interactions with CCR5, which can manifest *in vitro* as an increased ability to engage certain alternative coreceptors.

## Methods

### Ethics statement

As we have stated previously [8], written informed consent was provided by the subjects for the use of stored plasma samples from which the Env clones used here were derived . Ethics approval for the use of these samples was granted by the Medical Research Council of Zimbabwe (MRCZ/A/918) and by the Central Medical Scientific Ethics Committee of Denmark (624-01-0031).

### Cells

NP2-CD4 cells stably expressing either CCR5, CXCR4, CCR3, CCR8 or FPRL1 were maintained as described previously [35].

### Env plasmids

The Envs used in this study are expressed from the pSVIII-Env mammalian expression plasmid [36], and have been described in detail recently [8]. The Envs have been assigned GenBank accession numbers HQ707833 to HQ708154. The clinical details of the study subjects are also described in detail in Jakobsen et al [8]. Briefly, Envs were cloned directly from plasma of 21 ART-naïve individuals who progressed from chronic to advanced stages of C-HIV infection, as determined by significant declines in CD4+ T-cell count, over an approximately 3-year period. Stored plasma samples that were collected at study enrolment (“E”), approximately 1 year later (“I”, representing the “intermediate” sample), and approximately 3 years after enrolment (“F”, representing the “final” sample that was collected after CD4+ T-cell decline) were used for Env cloning. Between 2 and 8 functional and genotypically unique Envs from each plasma sample, totalling 300 Envs across the cohort, were identified based on the ability to support the entry of Env-pseudotyped GFP reporter viruses into JC53 cells [37]. The nomenclature of the Envs follows the “patient-sample-clone number” order. For example, Env clone “1109-F-30” is clone number 30 from the final plasma sample of subject 1109.

### Production of Env-pseudotyped luciferase reporter viruses

Env-pseudotyped, luciferase reporter viruses were produced by transfection of 293 T cells with pCMV∆P1∆envpA, pHIV-1Luc and pSVIII-Env plasmids at a ratio of 1:3:1 using Lipofectamine 2000 (Invitrogen), as described previously [34,38-43]. Supernatants were harvested 48 h later, clarified by filtration through 0.45 μM filters, aliquotted and stored at -80°C.

### HIV-1 entry assays

The ability of Env-pseudotyped luciferase reporter viruses to use CCR5, CXCR4, or the alternative coreceptors CCR3, CCR8 and FPRL1 was determined by single-round entry assays using NP2-CD4 cells stably expressing either coreceptor. Briefly, 1 × 10^4^ cells were inoculated with 5-fold dilutions of each virus preparation for 6-hours at 37°C. Cells were then media changed and incubated for 48-hours at 37°C. Virus entry was then measured by assaying luciferase activity in cell lysates (Promega), according to the manufacturers’ protocol. Usage of a particular coreceptor by a given virus was determined to be positive if it yielded a titratable luciferase signal above background. For correlative analyses of CCR5/alternative coreceptor usage, entry levels from a common dilution of virus that resulted in luciferase signals within the linear range of activity for each of the cell types that were positive for virus entry were analyzed. In most cases this was a 1:5 dilution. For viruses that used only CCR5, data from a 1:5 dilution was similarly selected for analysis provided this dilution resulted in linear-range luciferase signals. For cell types yielding no detectable entry for a given virus, background luciferase signals from the same dilution that yielded linear range luciferase signals in the respective permissive cell populations were used for analysis. The negative controls used to determine the background level of luciferase activity included mock-infected cells treated with culture medium instead of virus, and cells inoculated with luciferase reporter virus pseudotyped with the non-functional ΔKS Env [44].

### Env mutants

All gp120 mutants were synthesized by GenScript Pty. Ltd. (Piscataway, NJ, USA), and subcloned into the pSVIII-Env expression vector [8]. The authenticity of the gp120 mutants was verified by full-length sequencing. This series of mutants involved replacing various motifs of the X4 1109-F-30 Env with those of the antecedent R5 1109-E-30 Env from subject 1109. The amino acid sequences of the V1 and V3 loops of these 2 Envs, where the distinguishing amino acid changes were located, and the consequent amino acid sequences of the various 1109-F-30 Env mutants are shown in Figure 4. The Env mutants consist of the 1109-F-30 Env with Pro318 of the 1109-E-10 Env (M1), 1109-F-30 with deletion of the Ile314-Gly315 insertion (M2), 1109-F-30 with Pro318 of the 1109-E-10 Env and deletion of the Ile314-Gly315 insertion (M3), 1109-F-30 with Thr329 of 1109-E-10 (M4), 1109-F-30 with Asn331 of 1109-E-10 (M5), 1109-F-30 with the whole V1 loop of 1109-E-10 (M6), 1109-F-30 with the V1 loop and Pro318 of 1109-E-10 (M7), 1109-F-30 with the V1 loop of 1109-E-10 and deletion of the Ile314-Gly315 insertion (M8), 1109-F-30 with the V1 loop and Thr329 of 1109-E10 (M9), 1109-F-30 with the V1 loop and Asn331 of 1109-E-10 (M10), 1109-F-30 with the V1 loop and Pro318 of 1109-E-10 and deletion of the Ile314-Gly315 insertion (M11), 1109-F-30 with the V1 loop, Pro318 and Thr329 of 1109-E-10 (M12), and 1109-F-30 with the V1 loop, Pro318 and Asn331 of 1109-E-10 (M13). In addition, we produced an Env mutant of 1109-F-30 containing the whole V3 loop of 1109-E-10 (M14), and a mutant of 1109-F-30 containing both the V3 and V1 loops of 1109-E-10 (M15).

## Competing interests

The authors declare that they have no competing interests.

## Authors’ contributions

KC, MRJ, JS, MR, AE, JKF and KB performed the experiments. MG performed statistical analyses. KC, MRJ and PRG designed the experiments. KC, MRJ, MJC and PRG interpreted the results. KC and PRG wrote the manuscript. All authors helped edit the manuscript and have read and approved the final version.

## Supplementary Material

Additional file 1: Table S1 Alternative coreceptor usage of Env clones. The Level of virus entry in NP2-CD4 cells expressing CCR3, FPRL1 or CCR8 or was scored as – (<5 fold above background), + (5-50 fold above background), ++ (50-300 fold above background), or +++ (>300 fold above background). The results for virus entry into NP2-CD4 cells expressing CCR5 or CXCR4, using equivalent infectious units of virus inoculum, are shown alongside these data for comparison. E, I and F refer to Envs cloned from plasma obtained at study enrolment, approximately 1 year later (intermediate), and approximately 3 years after enrolment (Final), respectively (Jakobsen et al., [8]). Click here for file
