# Supplementary material for: Linkages between HIV-1 specificity for CCR5 or CXCR4 and in vitro usage of alternative coreceptors during progressive HIV-1 subtype C infection
Source: Retrovirology. 2013 Sep 16;10:98. doi: 10.1186/1742-4690-10-98 (PMC3849974; doi:10.1186/1742-4690-10-98)
Supplement: Additional file 1: Table S1 — Alternative coreceptor usage of Env clones. The Level of virus entry in NP2-CD4 cells expressing CCR3, FPRL1 or CCR8 or was scored as – (<5 fold above background), + (5-50 fold above background), ++ (50-300 fold above background), or +++ (>300 fold above background). The results for virus entry into NP2-CD4 cells expressing CCR5 or CXCR4, using equivalent infectious units of virus inoculum, are shown alongside these data for comparison. E, I and F refer to Envs cloned from plasma obtained at study enrolment, approximately 1 year later (intermediate), and approximately 3 years after enrolment (Final), respectively (Jakobsen et al., [8]). [file 1742-4690-10-98-S1.pdf]

Supplementary Information

Linkages between HIV-1 specificity for CCR5 or CXCR4 and *in vitro* usage of alternative coreceptors during progressive HIV-1 subtype C infection

Kieran Cashin[1,3], Martin R. Jakobsen[4], Jasminka Sterjovski[1,5], Michael Roche[1,5], Anne Ellett[1], Jacqueline K. Flynn[1,5], Katharina Borm[1,8], Maelenn Gouillou[2], Melissa J. Churchill[1,6,7], and Paul R. Gorry[1,3,5*].

Centers for Biomedical Research[1] and Population Health[2], Burnet Institute, Melbourne, Australia;

[3]Department of Microbiology and Immunology, University of Melbourne, Australia;

[4]Department of Biomedicine, Aarhus University, Aarhus, Denmark;

Departments of Infectious Diseases[5], Microbiology[6] and Medicine[7], Monash University, Australia;

[8]Department of Microbiology, La Trobe University, Melbourne, Victoria, Australia.

***Corresponding Author**

Paul R. Gorry, PhD.

Center for Biomedical Research, Burnet Institute, 85 Commercial Rd, Melbourne, 3004, Victoria, Australia.

Ph: +61-3-9282-2129

Email: gorry@burnet.edu.au

Table S1 (part 1). Alternative coreceptor usage of Env clones.

Patient ID	Genbank number	Env clone		Coreceptor usage				
		T	#	CCR5	CXCR4	CCR3	FPRL-1	CCR8
204	HQ708023	E	7	++	-	+	++	-
	HQ708019	E	10	+	-	-	+	-
	HQ708020	E	12	+	-	-	+	-
	HQ708021	E	15	++	-	-	++	-
	HQ708022	E	17	+	-	+	-	-
	HQ708032	I	6	+	-	-	+	-
	HQ708033	I	9	++	-	-	+++	-
	HQ708031	I	10	+	-	-	-	-
	HQ708025	F	3	+++	-	++	+++	-
	HQ708026	F	4	+	-	-	+	-
	HQ708027	F	5	+++	-	+	++	-
	HQ708029	F	8	+++	-	-	++	-
	HQ708024	F	10	+	-	-	-	-
258	HQ708049	E	3	++	-	-	+	-
	HQ708050	E	5	+++	-	++	+++	-
	HQ708051	E	6	+++	-	+	++	-
	HQ708047	E	20	+++	-	+	+++	-
	HQ708048	E	23	+++	-	+	+++	-
	HQ708058	I	1	++	-	+	++	-
	HQ708059	I	2	+++	-	++	+++	-
	HQ708060	I	4	+	-	++	+++	-
	HQ708061	I	6	++	-	++	+++	-
	HQ708062	I	9	+	-	++	+++	-
	HQ708052	F	1	+++	-	-	+++	-
	HQ708053	F	2	+++	-	-	++	-
	HQ708057	F	7	+++	-	+++	+++	-
	HQ708054	F	20	+++	-	+	+++	-
	HQ708055	F	22	+	-	-	+	-
	HQ708056	F	24	+++	-	+	+++	-
455	HQ708063	E	23	+++	-	+	+	+
	HQ708064	E	24	+++	-	+	++	-
	HQ708065	E	27	+++	-	+	++	-
	HQ708066	E	28	++	-	-	+	-
	HQ708073	I	2	+++	-	+++	+	-
	HQ708076	I	3	+++	-	++	+	-
	HQ708077	I	5	+++	-	+++	+	+
	HQ708078	I	9	+++	-	++	+	-
	HQ708074	I	22	+++	-	++	+	-
	HQ708075	I	24	+++	-	++	++	-
	HQ708067	F	1	+++	-	++	+	-
	HQ708068	F	2	+++	-	-	++	-
	HQ708071	F	5	++	-	-	+	-
	HQ708072	F	9	+++	-	++	+	-
	HQ708069	F	22	+++	-	++	+	-
	HQ708070	F	23	+++	-	-	+	-
513	HQ708080	E	4	+	-	-	-	-
	HQ708080	E	8	+++	-	++	-	-
	HQ708089	I	1	+	-	-	-	-
	HQ708093	I	5	+++	-	++	+	-
	HQ708094	I	8	+++	-	+	-	-
	HQ708090	I	16	++	-	+	-	-
	HQ708092	I	21	+	-	-	-	-
	HQ708082	F	1	++	-	++	-	-
	HQ708083	F	2	+	-	-	-	-
	HQ708087	F	4	++	-	+	+	+
	HQ708092	F	7	++	-	+	+	+
	HQ708092	F	21	++	-	+	+	+
	HQ708084	F	22	++	-	+	+	+
	HQ708085	F	26	++	-	+	+	+
	HQ708086	F	27	+	-	+	+	+

Table S1 (part 2). Alternative coreceptor usage of Env clones.

Patient ID	Genbank number	Env clone		Coreceptor usage				
		T	#	CCR5	CXCR4	CCR3	FPRL-1	CCR8
550	HQ708097	E	3	+++	-	++	++	-
	HQ708098	E	8	+++	-	+	+++	-
	HQ708095	E	13	+++	-	+++	+++	-
	HQ708096	E	20	+	-	-	-	-
	HQ708104	I	2	+++	-	+++	+++	-
	HQ708108	I	7	+++	-	+++	+++	-
	HQ708105	I	30	+++	-	-	+++	-
	HQ708106	I	31	+++	-	+	+++	-
	HQ708107	I	32	+++	-	-	+++	-
	HQ708099	F	1	+++	-	+	+++	-
	HQ708102	F	7	+++	-	++	+++	-
	HQ708103	F	9	+++	-	+	+++	-
	HQ708100	F	12	+++	-	+++	+++	-
	HQ708101	F	20	+++	-	++	+++	-
574	HQ708112	E	5	+	-	+	+	+
	HQ708113	E	6	+++	-	+	++	+
	HQ708109	E	11	++	-	+	+	+
	HQ708126	I	4	+++	-	+	+	+
	HQ708122	I	12	+++	-	-	++	-
	HQ708123	I	13	++	-	-	-	-
	HQ708124	I	14	+++	-	-	+	-
	HQ708127	I	15	++	-	-	+	-
	HQ708114	F	1	+++	-	+	+	-
	HQ708118	F	4	++	-	+	+	-
	HQ708119	F	5	++	-	+	+	-
	HQ708120	F	6	+	-	-	-	-
	HQ708121	F	8	+++	-	++	++	+
	HQ708115	F	10	++	-	+	+	-
	HQ708116	F	12	++	-	-	+	-
	HQ708117	F	15	++	-	+	+	-
805	HQ708127	E	15	+++	-	++	+++	-
	HQ708128	E	29	++	-	+	++	-
	HQ708129	E	31	++	-	-	++	-
	HQ708130	I	2	+++	-	+	++	-
	HQ708132	I	7	++	-	-	++	-
	HQ708133	I	8	++	-	+	+++	-
	HQ708131	I	34	++	-	+	++	-
858	HQ708135	E	1	+++	-	+	+++	-
	HQ708136	E	2	+++	-	-	+++	-
	HQ708141	E	4	+++	-	-	++	-
	HQ708142	E	8	+++	-	-	+++	-
	HQ708137	E	22	+++	-	-	-	-
	HQ708138	E	23	+++	-	+	+++	-
	HQ708140	E	38	+++	-	+	+++	-
	HQ708149	I	1	+++	-	-	++	-
	HQ708152	I	5	+++	-	-	+++	-
	HQ708153	I	7	++	-	-	+	-
	HQ708154	I	8	+++	-	+	+++	-
	HQ708150	I	10	+++	-	-	-	-
	HQ708151	I	20	+++	-	-	++	-
	HQ708143	F	1	+	-	-	-	-
	HQ708146	F	3	+++	-	-	-	-
	HQ708147	F	6	+++	-	++	++	-
	HQ708148	F	9	+++	-	+	+++	-
	HQ708134	F	21	+++	-	+	+	-
	HQ708144	F	26	+++	-	-	++	-
	HQ708145	F	27	+	-	-	-	-

<u>Table S1 (part 3).</u> Alternative coreceptor usage of Env clones.

Patient ID	Genbank number	Env clone		Coreceptor usage				
		T	#	CCR5	CXCR4	CCR3	FPRL-1	CCR8
1109	HQ707833	E	10	+++	-	+	+++	+
	HQ707834	E	42	+++	-	++	+++	++
	HQ707835	E	43	++	-	-	+++	-
	HQ707836	E	46	++	-	-	++	-
	HQ707837	E	49	++	-	-	+	+
	HQ707848	I	3	++	-	+	+++	+
	HQ707849	I	9	++	-	-	++	+
	HQ707844	I	10	+++	-	++	+++	+
	HQ707847	I	19	+++	-	++	+++	++
	HQ707838	F	30	-	+++	+++	+	+
	HQ707839	F	31	-	+++	+++	+	++
	HQ707840	F	33	-	+++	++	-	-
	HQ707841	F	34	-	+++	+++	+	++
	HQ707842	F	38	-	+++	+++	-	++
	HQ707843	F	39	-	+++	+++	-	++
1114	HQ707850	E	1	++	-	-	-	-
	HQ707853	E	3	+++	-	+++	++	+++
	HQ707854	E	8	+++	-	-	-	-
	HQ707855	E	9	+++	-	-	-	++
	HQ707851	E	10	++	-	-	-	-
	HQ707861	I	1	+++	-	-	-	-
	HQ707863	I	4	+++	-	++	+	+++
	HQ707864	I	5	+++	-	++	+	++
	HQ707865	I	6	+++	-	++	+	+++
	HQ707866	I	8	+++	-	+	-	++
	HQ707862	I	10	++	-	+	-	+
	HQ707856	F	17	+++	-	-	-	-
	HQ707857	F	18	+++	-	-	-	-
	HQ707858	F	19	+++	-	+	-	+
	HQ707859	F	33	+++	-	-	-	-
	HQ707860	F	37	+	-	-	-	-
1136	HQ707867	E	1	+++	-	-	++	++
	HQ707870	E	4	+++	-	-	++	+
	HQ707868	E	11	+++	-	-	+++	-
	HQ707869	E	12	+++	-	-	+++	+
	HQ707880	I	3	+++	-	-	++	++
	HQ707881	I	4	+++	-	-	+++	+
	HQ707877	I	10	+++	-	-	+++	+
	HQ707878	I	11	+++	-	-	+++	-
	HQ707879	I	12	+++	-	-	+++	+
	HQ707875	F	5	+++	-	-	++	-
	HQ707876	F	7	+++	-	-	-	-
	HQ707871	F	11	+++	-	++	+++	+++
	HQ707872	F	12	+++	-	++	+++	+++
	HQ707873	F	13	+++	-	+++	+++	+++
1375	HQ707882	E	2	+++	-	+	+++	++
	HQ707885	E	3	+++	-	-	++	-
	HQ707886	E	5	+++	-	+	++	+
	HQ707887	E	7	+++	-	+	+++	+
	HQ707883	E	20	+++	-	+	+++	+
	HQ707884	E	21	+++	-	-	+++	-
	HQ707891	I	1	+++	-	+	+++	-
	HQ707892	I	2	+++	-	++	+++	+
	HQ707893	I	3	+++	-	++	+++	+
	HQ707894	I	6	+++	-	++	+++	++
	HQ707895	I	8	+++	-	+	+++	+
	HQ707896	I	9	+++	-	++	+++	+
	HQ707888	F	13	+++	-	+	++	-
	HQ707889	F	20	+++	-	+	+++	-
	HQ707890	F	21	++	-	-	++	-

Table S1 (part 4). Alternative coreceptor usage of Env clones.

Patient ID	Genbank number	Env clone		Coreceptor usage				
		T	#	CCR5	CXCR4	CCR3	FPRL-1	CCR8
1408	HQ707897	E	30	+++	-	-	+	-
	HQ707898	E	31	+++	-	+	++	+
	HQ707899	E	33	+++	-	++	+++	+
	HQ707900	E	34	+++	-	-	++	+
	HQ707901	E	35	+++	-	+	+	-
	HQ707902	E	40	+++	-	-	++	-
	HQ707903	I	1	+++	-	+	+	-
	HQ707904	I	31	+++	-	+	+	-
	HQ707905	I	33	+++	-	+	+	-
	HQ707906	I	34	+++	-	+	+	-
	HQ707907	I	35	+++	-	++	++	-
	HQ707908	I	36	+++	-	+	+	-
	HQ707909	I	37	+	-	-	+	-
	HQ707910	I	38	+++	-	-	-	-
1441	HQ707911	E	1	+++	-	-	++	+
	HQ707912	E	2	+++	-	-	++	+
	HQ707915	E	6	+++	-	+	+++	+
	HQ707916	E	7	+++	-	-	++	-
	HQ707913	E	24	+	-	-	++	-
	HQ707914	E	26	+	-	-	++	-
	HQ707921	I	2	+++	-	+	++	++
	HQ707922	I	3	+++	-	-	++	-
	HQ707923	I	8	+++	-	-	+++	-
	HQ707924	I	9	+++	-	+	+++	++
	HQ707917	F	20	+++	-	++	+++	+
	HQ707918	F	24	+++	-	-	++	+
	HQ707919	F	28	+++	-	+	++	+
	HQ707920	F	32	+++	-	++	++	++
1503	HQ707925	E	11	+++	-	-	-	-
	HQ707926	E	13	++	-	-	-	-
	HQ707928	E	22	+++	-	-	-	-
	HQ707943	I	7	+++	-	+	+	-
	HQ707939	I	11	+++	-	++	+++	++
	HQ707940	I	13	+++	-	+	-	-
	HQ707941	I	24	+	-	-	-	-
	HQ707942	I	27	+++	-	+	+	-
	HQ707934	F	6	+	-	-	+	-
	HQ707935	F	7	+++	-	-	-	-
	HQ707936	F	8	+++	-	+++	+++	++
	HQ707937	F	9	+++	-	-	-	-
	HQ707931	F	40	+++	-	++	+++	++
	HQ707932	F	42	+++	-	++	++	++
	HQ707933	F	43	+++	-	-	-	-
1554	HQ707945	E	2	+++	-	+++	++	++
	HQ707946	E	3	++	-	++	-	-
	HQ707950	E	8	+++	-	+++	++	++
	HQ707944	E	10	+++	-	+++	++	++
	HQ707959	I	6	+++	-	++	++	++
	HQ707960	I	7	++	-	+	-	-
	HQ707961	I	8	+++	-	-	++	-
	HQ707962	I	9	+++	-	-	++	-
	HQ707955	F	3	++	-	-	-	-
	HQ707956	F	4	+++	-	-	++	-
	HQ707957	F	8	+++	-	++	+	+
	HQ707951	F	10	+++	-	++	+++	+
	HQ707952	F	25	+++	-	++	+	+
	HQ707953	F	26	+++	-	+++	+	+
	HQ707954	F	29	+++	-	+++	++	++

Table S1 (part 5). Alternative coreceptor usage of Env clones.

Patient ID	Genbank number	Env clone		Coreceptor usage				
		T	#	CCR5	CXCR4	CCR3	FPRL-1	CCR8
1684	HQ707964	E	2	+	-	-	-	-
	HQ707965	E	7	+++	-	++	+++	+
	HQ707966	E	9	+++	-	-	++	-
	HQ707963	E	19	+++	-	++	+++	-
	HQ707972	I	13	++	-	+	++	-
	HQ707973	I	26	++	-	-	+	-
	HQ707974	I	29	+++	-	+	+	-
	HQ707975	I	30	++	-	+	+	-
	HQ707971	F	8	+++	-	++	+++	-
	HQ707967	F	12	+	-	-	-	-
	HQ707968	F	17	+++	-	-	+++	-
	HQ707969	F	22	+++	-	+++	+++	++
1689	HQ707976	E	5	+	-	-	-	-
	HQ707977	E	6	++	-	-	-	-
	HQ707978	E	8	++	-	-	-	-
	HQ707979	E	9	++	-	-	-	-
	HQ707989	I	7	++	-	-	-	-
	HQ707984	I	10	++	-	-	-	-
	HQ707985	I	24	++	-	-	-	-
	HQ707986	I	25	++	-	-	-	-
	HQ707987	I	26	++	-	-	-	-
	HQ707988	I	28	++	-	-	-	-
	HQ707980	F	2	+	-	-	-	-
	HQ707983	F	5	++	-	-	-	-
	HQ707981	F	25	+	-	-	-	-
	HQ707982	F	26	+	-	-	-	-
1854	HQ707991	E	5	+	-	-	-	-
	HQ707992	E	8	+++	-	++	+++	++
	HQ707990	E	10	+++	-	+++	+++	++
	HQ708001	I	3	+	-	-	-	-
	HQ708002	I	4	+++	-	+++	+++	+
	HQ708005	I	6	++	-	+	+	-
	HQ708006	I	7	+++	-	++	+	-
	HQ708000	I	20	+++	+	+++	+++	++
	HQ707997	F	4	+++	+	+++	+++	+++
	HQ707998	F	6	+++	-	++	++	++
	HQ707999	F	8	+++	-	++	++	+
	HQ707993	F	14	++	-	-	-	-
	HQ707994	F	17	++	-	-	-	-
	HQ707995	F	18	++	-	+	+	-
	HQ707996	F	21	+++	-	++	++	-
2042	HQ708007	E	15	++	-	-	+	-
	HQ708008	E	25	+++	-	-	+	+
	HQ708009	E	26	+++	-	-	++	-
	HQ708014	I	13	++	-	-	+	-
	HQ708015	I	21	+++	-	+	++	-
	HQ708016	I	27	++	-	-	-	-
	HQ708017	I	38	++	-	+	++	-
	HQ708010	F	24	+++	-	+	++	-
	HQ708011	F	25	++	-	-	-	-
	HQ708012	F	26	+	-	-	-	-
	HQ708013	F	28	+++	-	-	++	-
2253	HQ708037	E	21	+++	-	+	++	++
	HQ708038	E	22	+++	-	-	-	-
	HQ708039	E	23	+++	-	+	++	+
	HQ708040	E	24	+++	-	+	++	++
	HQ708041	I	21	+++	-	+	-	+
	HQ708042	I	22	++	-	-	-	-
	HQ708043	I	23	+++	-	+	+	++
	HQ708044	I	27	++	-	-	-	-
	HQ708045	I	28	++	-	-	-	-
	HQ708046	I	32	++	-	-	+	+
	HQ708034	F	21	+++	-	+	+	-
	HQ708035	F	29	++	-	-	-	-
	HQ708036	F	36	+++	-	+	+	+

Legend for Table S1. The level of virus entry in NP2-CD4 cells expressing CCR3, FPRL1 or CCR8 or was scored as – (< 5 fold above background), + (5-50 fold above background), ++ (50-300 fold above background), or +++ (>300 fold above background). The results for virus entry into NP2-CD4 cells expressing CCR5 or CXCR4, using equivalent infectious units of virus inoculum, are shown alongside these data for comparison. E, I and F refer to Envs cloned from plasma obtained at study enrolment, approximately 1 year later (intermediate), and approximately 3 years after enrolment (Final), respectively (Jakobsen et al., 2013).
